# Supplementary material for: Impact of Dietary Arachidonic Acid on Gut Microbiota Composition and Gut–Brain Axis in Male BALB/C Mice
Source: Nutrients. 2022 Dec 15;14(24):5338. doi: 10.3390/nu14245338 (PMC9786182; doi:10.3390/nu14245338)
Supplement: Supplementary file 1 [file nutrients-14-05338-s001.zip › nutrients-2087598-supplementary.pptx]

## Slide 1
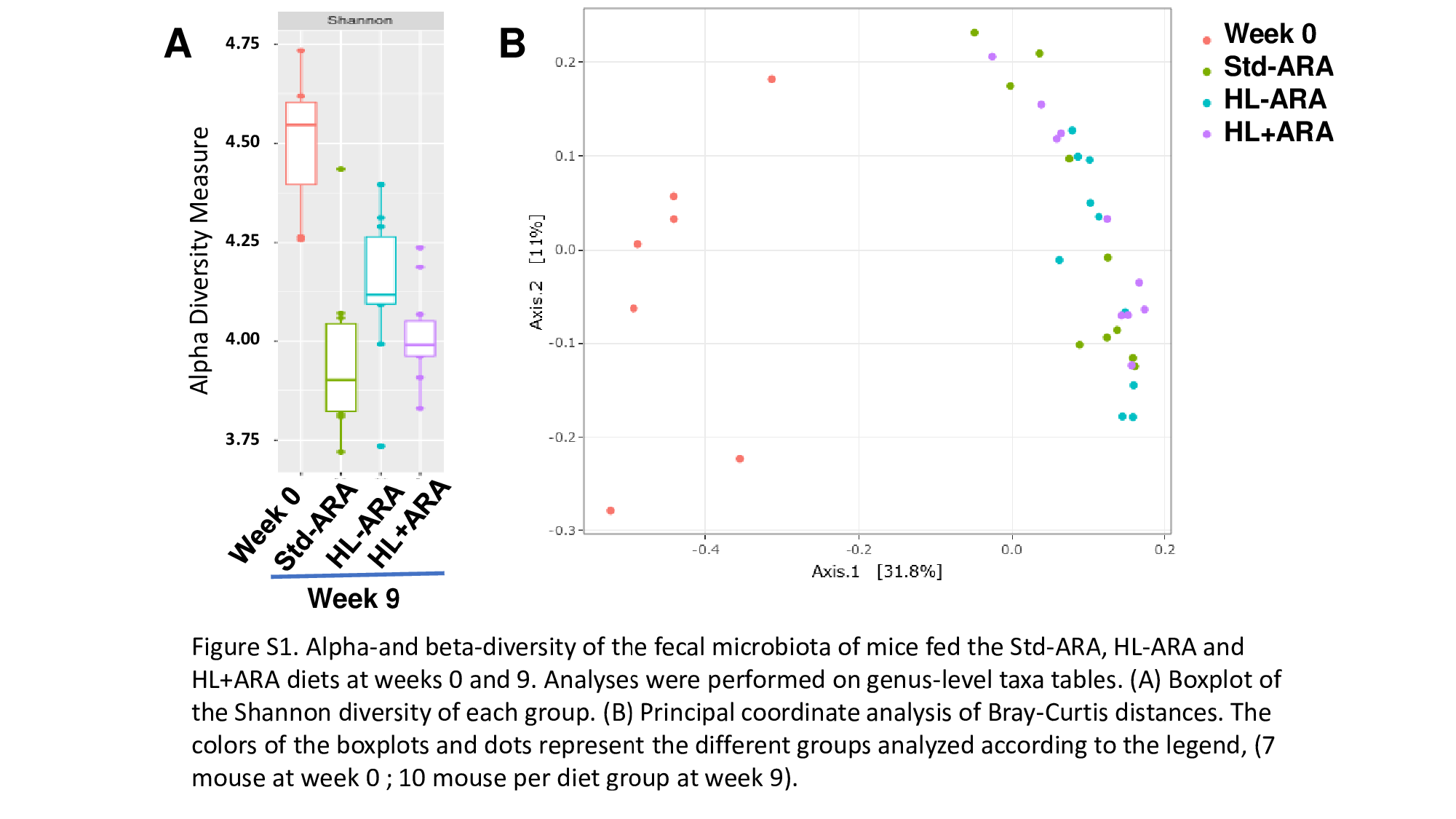

## Slide 2
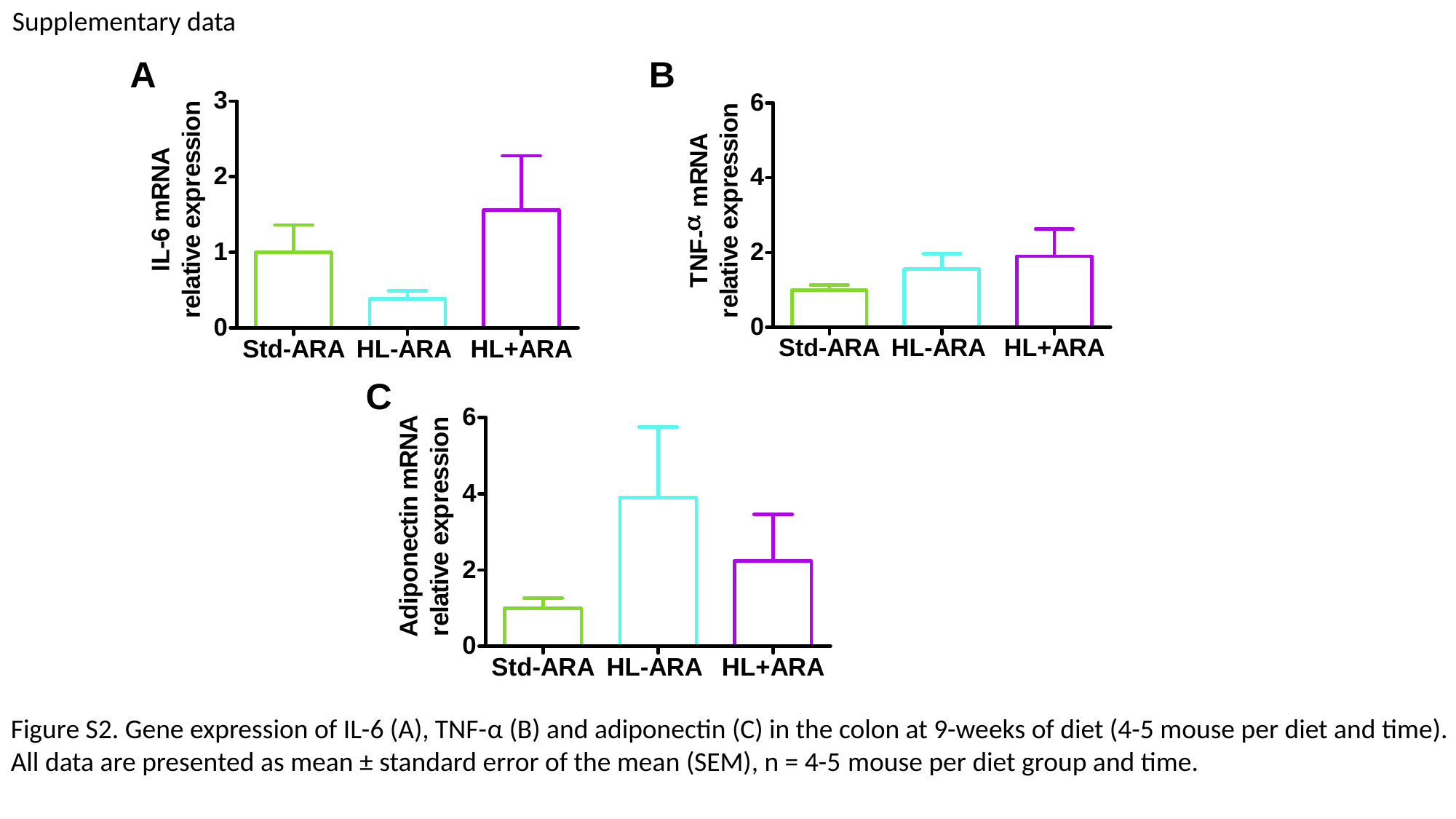

Supplementary data
A
B
C
Figure S2. Gene expression of IL-6 (A), TNF-α (B) and adiponectin (C) in the colon at 9-weeks of diet (4-5 mouse per diet and time). All data are presented as mean ± standard error of the mean (SEM), n = 4-5 mouse per diet group and time.

## Slide 3
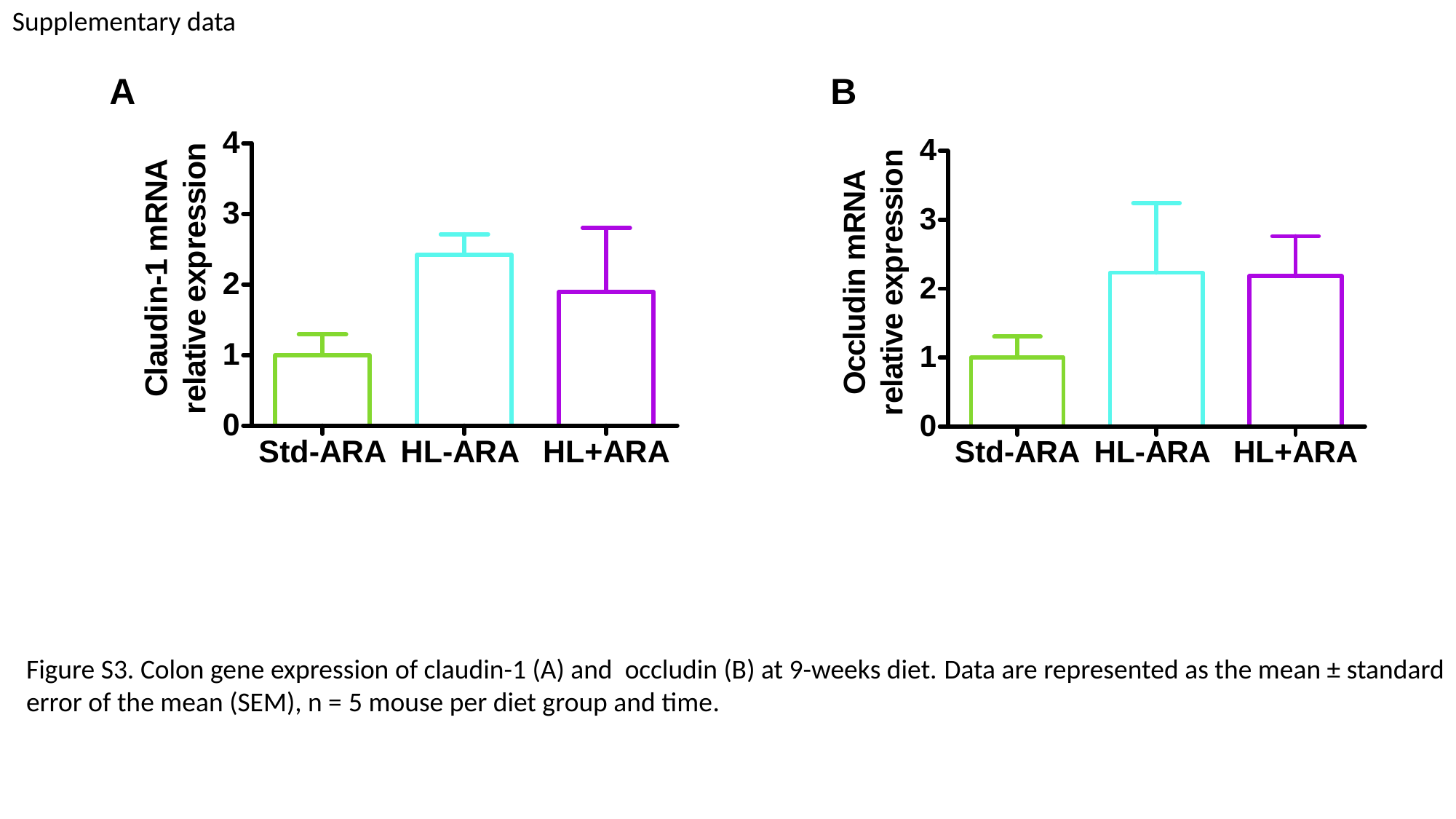

Supplementary data
A
B
Figure S3. Colon gene expression of claudin-1 (A) and occludin (B) at 9-weeks diet. Data are represented as the mean ± standard error of the mean (SEM), n = 5 mouse per diet group and time.

## Slide 4
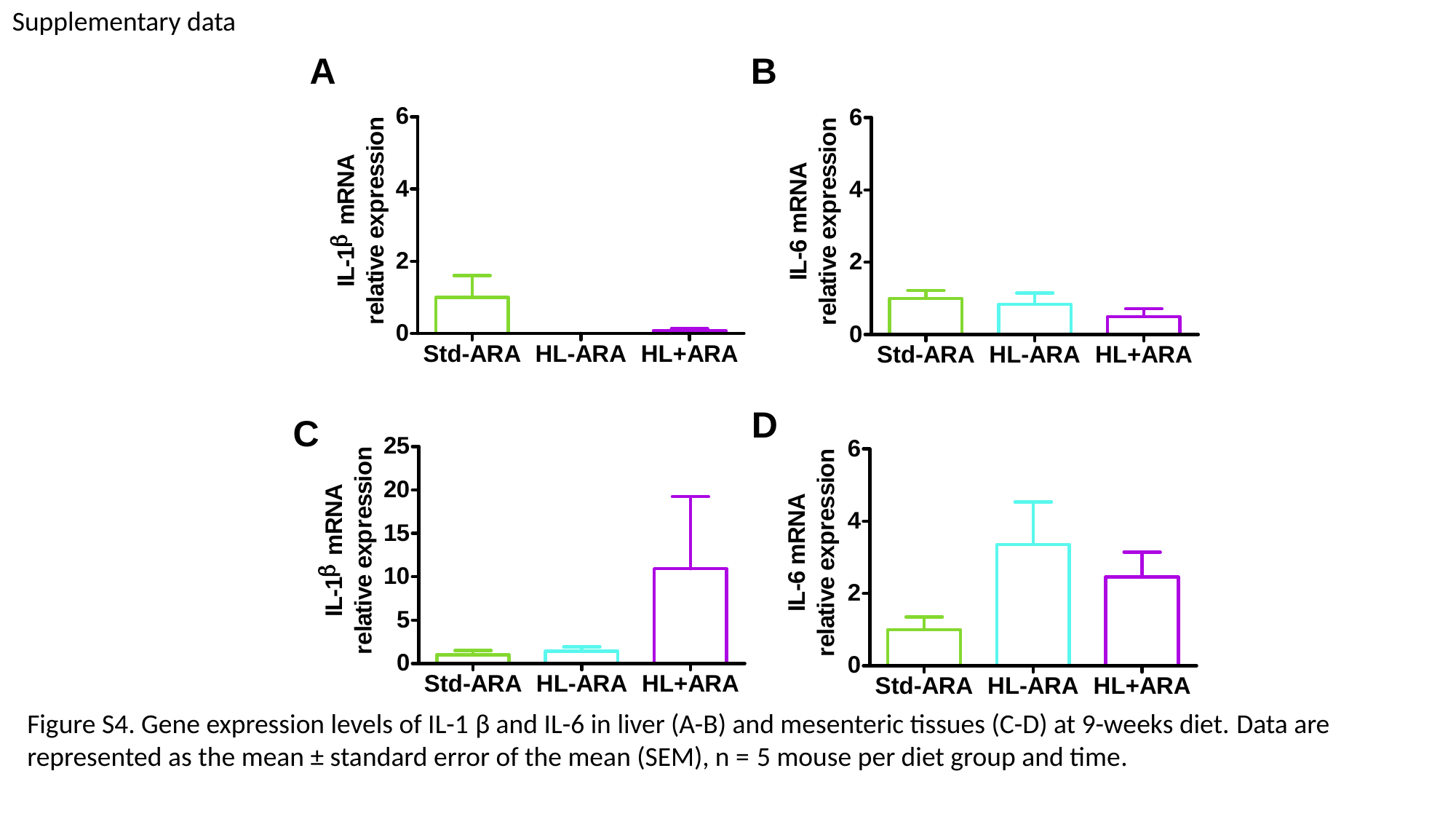

Supplementary data
A
B
D
C
Figure S4. Gene expression levels of IL-1 β and IL-6 in liver (A-B) and mesenteric tissues (C-D) at 9-weeks diet. Data are represented as the mean ± standard error of the mean (SEM), n = 5 mouse per diet group and time.

## Slide 5
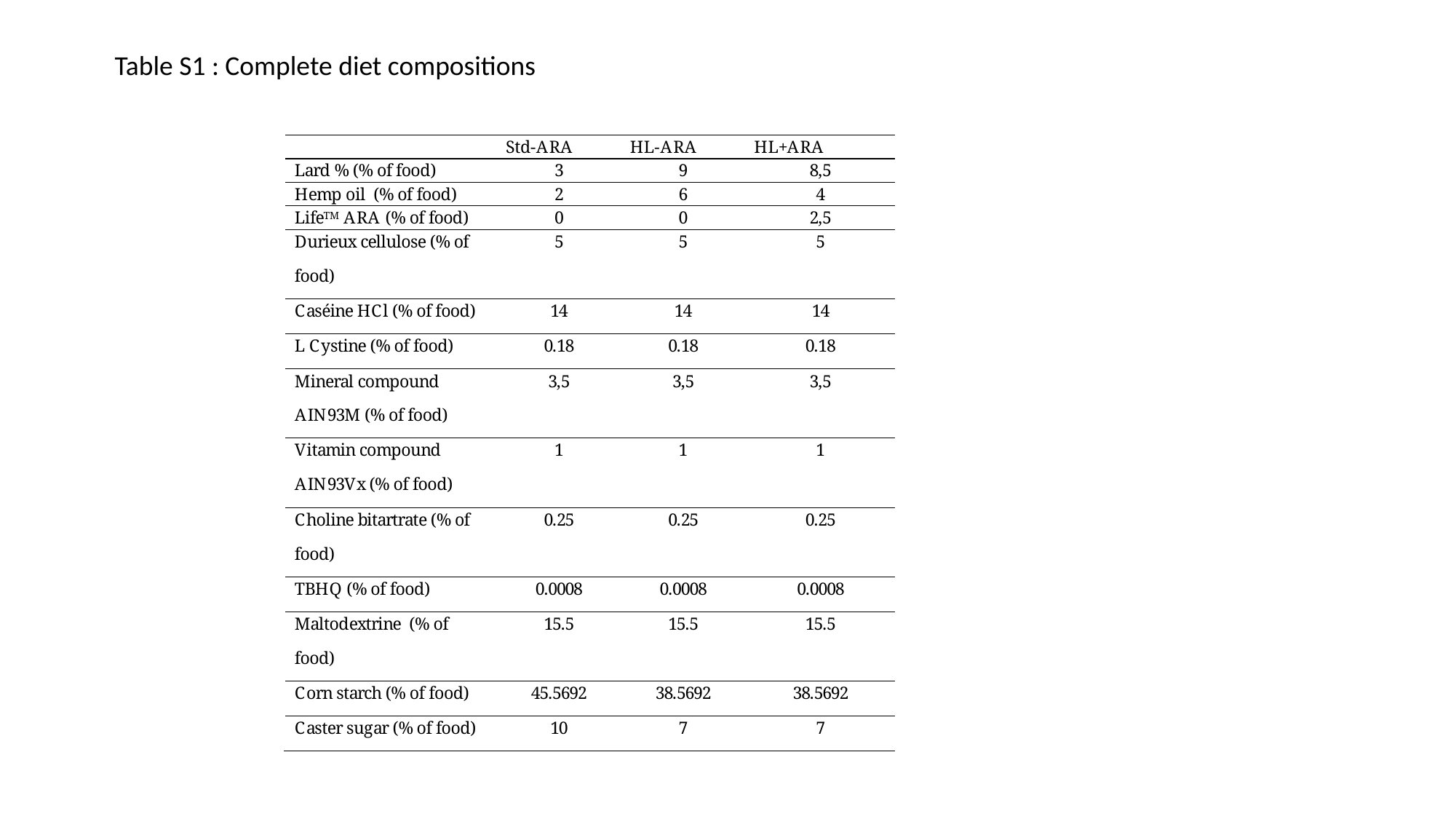

Table S1 : Complete diet compositions
